# No Relationship between Maternal Iron Status and Postpartum Depression in Two Samples in China

**DOI:** 10.1155/2012/521431

**Published:** 2012-07-30

**Authors:** Rinat Armony-Sivan, Jie Shao, Ming Li, Gengli Zhao, Zhengyan Zhao, Guobing Xu, Min Zhou, Jianying Zhan, Yang Bian, Chai Ji, Xing Li, Yaping Jiang, Zhixiang Zhang, Blair J. Richards, Twila Tardif, Betsy Lozoff

**Affiliations:** ^1^Center for Human Growth and Development, University of Michigan, Ann Arbor, MI 48109, USA; ^2^Department of Psychology, Ashkelon Academic College, 78109 Ashkelon, Israel; ^3^Department of Child Health Care, Children's Hospital Zhejiang University School of Medicine, Hangzhou 310003, China; ^4^Department of Pediatrics, Peking University First Hospital, Beijing 100034, China; ^5^Women's and Children's Health Center, Peking University, Beijing 100034, China; ^6^Department of Clinical Laboratory, Peking University First Hospital, Beijing 100034, China; ^7^Department of Psychology, University of Michigan, Ann Arbor, MI 48109, USA; ^8^Department of Pediatrics and Communicable Diseases, University of Michigan, Ann Arbor, MI 48109, USA

## Abstract

Maternal iron status is thought to be related to postpartum depressive symptoms. The purpose of the present study was to evaluate the relationship between pre- and postnatal maternal iron status and depressive symptoms in pilot (*n* = 137) and confirmatory (*n* = 567) samples of Chinese women. Iron status was evaluated at mid- and late pregnancy and 3 days postpartum. The Edinburgh Postnatal Depression Scale (EPDS) was used to assess maternal postpartum depression 24–48 hours after delivery and 6 weeks later. In the pilot sample, correlations between early- and late-pregnancy maternal Hb and EPDS scores at 6 weeks were *r* = 0.07 and −0.01, respectively (nonsignificant). In the confirmatory sample, the correlations between maternal iron measures (Hb, MCV, ZPP, ferritin, sTfR, and sTfR Index) in mid- or late pregnancy or 3 days postpartum and EPDS scores shortly after delivery or at 6 weeks were also low (*r* values < 0.10). EPDS scores in anemic and nonanemic mothers did not differ, regardless of sample or timing of maternal iron status assessment. In addition, women with or without possible PPD were similar in iron status in both samples. Thus, there was no relationship between maternal iron status and postpartum depression in these samples.

## 1. Introduction

Iron deficiency anemia is a global public health problem affecting both developing and developed countries with major consequences for human health as well as social and economic development. Although direct estimates of the worldwide prevalence of iron deficiency are problematic [1], anemia—a late manifestation of iron deficiency—affects an estimated 30% of nonpregnant women of reproductive age and 42% of pregnant women [2].

Iron deficiency in women has been related to fatigue and poorer general health [3–5] and emotional and cognitive function [6–11]. However, the relationship between postpartum depression (PPD) and maternal iron status remains unclear. One of the earliest studies reported that low Hb was associated with such postnatal symptoms as low energy, faintness/dizziness, painful perineal sutures, and tingling of fingers and toes but not with PPD [12]. In contrast, several subsequent studies found that anemia and/or iron deficiency were associated with increased symptoms of postpartum blues and depression [8, 13–15]. Although postpartum depression was assessed in most of these studies by the Edinburgh Postnatal Depression Scale (EPDS) [16], differences in iron measures make direct comparisons difficult. Some studies focused on anemia [12–14], while others considered iron deficiency anemia (low Hb and at least two other iron deficiency parameters) [8] or iron deficiency based on low ferritin concentration [15]. Furthermore, relations between iron status and postpartum depression were analyzed in disparate ways. Some studies compared depressive symptoms in women with low versus high iron status [8, 14] others compared iron status in women with and without PPD symptoms [15]. Studies also differed in when maternal iron status was assessed, for example, during pregnancy, at delivery, or postpartum (see Table 1). 

The purpose of this study was to assess the relationship between prenatal and postnatal maternal iron status and depressed mood. We hypothesized that lower maternal iron status would be associated with more maternal postpartum symptoms of depression. 

## 2. Materials and Methods

This observational study was conducted in two phases: pilot and confirmatory. Both phases used data from ongoing longitudinal studies in China of developmental effects of iron deficiency in early life (supported by US NIH P01 HD39386 and R01 HD52069, B. Lozoff, Principal Investigator), one of which is conducted in conjunction with a study of iron supplementation in pregnancy (supported by a grant from Vifor Pharma, G. Zhao, Principal Investigator). The pilot sample consisted of 137 mothers from a rural area in Zhejiang province, in southeastern China. Because this sample was relatively small and Hb was the only measure of maternal iron status, we added a confirmatory sample with large *n* and a full panel of measures of maternal iron status. The confirmatory sample consisted of 567 mothers from a rural area in Hebei province in northern China. The studies were approved by the appropriate ethics committees of the University of Michigan and Children's Hospital Zhejiang University School of Medicine or Peking University First Hospital. Signed informed consent was obtained at enrollment. 

### 2.1. Participants and Data Collected

 Recruitment in both pilot and confirmatory studies was based on convenience samples. The participants were healthy pregnant women, 18 years or older, with a singleton pregnancy and no major complications. Women in the pilot sample were recruited during a routine prenatal visit at about 36 weeks of gestation at Fuyang Women and Children's Hospital, from May 2008 to January 2011. Hb concentrations in early and late pregnancy were obtained by chart review. To have conditions similar to the pilot sample, the confirmatory sample was drawn from the no-iron supplementation arm of an ongoing randomized clinical trial of supplementing women with folic acid with or without iron during pregnancy. Women were recruited at the initial prenatal visit between 13 and 20 weeks of gestation at Sanhe City Maternity and Child Health Institute, from November 2009 to November 2011. A complete blood count, ZPP (zinc protoporphyrin), serum ferritin, and sTfR (soluble transferrin receptor) were assessed. sTfR Index was calculated by dividing sTfR values (nmol/L) by the log_10_ of the ferritin concentration (ng/mL) [17, 18]. Maternal iron status was evaluated again in late pregnancy at about 36 weeks of gestation. For a subset of the confirmatory sample, Hb and MCV concentrations at 3 days postpartum were available from chart review. Anemia was defined as Hb < 110 g/L [2, 19], and high values of sTfR Index were considered indicative of iron deficiency. A sTfR Index cutoff point of 14 was used as recommended by the manufacture for clinical populations (>80% sensitivity and specificity; Beckman Coulter) [18]. In both studies, health providers used routine clinical practices for treating anemia in pregnancy, such as recommending increased dietary iron intake and iron supplements. In keeping with common practice in China [20–22], over 60% of births in both samples were by elective Caesarian section.

In both samples, depressive symptoms were assessed at 6 weeks postpartum using a Chinese version of the Edinburgh Postnatal Depression Scale (EPDS) [23]. The EPDS is a 10-item self-report scale widely used to screen for PPD [16]. The tester gave the instructions to the mother and made sure the mother understand them before filling out the form. If the motherrequested, the tester read the questions and/or explained them to her. Similar to previous studies, a total score of 10 or higher was considered as probable PPD [15, 23, 24]. In the confirmatory sample, the EPDS was also administered 24–48 hours postpartum. Reliability analysis of the EPDS (internal consistency) yielded Cronbach's *α* of 0.71 in the pilot study and 0.79 shortly after delivery and 0.78 at 6 weeks in the confirmatory study.

### 2.2. Statistical Analysis

 Background characteristics, Hb concentrations, and EPDS scores were compared in the pilot and confirmatory samples using *t*-tests for continuous variables and Fisher's exact analyses for categorical variables. Repeated measures analyses of variance were used to compare iron status in early/mid- and late-pregnancy and 3 days postpartum in each sample and EPDS score at 24–48 hours and 6 weeks after delivery in the confirmatory sample.

Correlations between iron measures and EPDS scores were performed for each sample. Given that EPDS and several iron measures were not normally distributed, nonparametric correlations were used (Spearman's rho). EPDS total scores were compared in anemic and nonanemic women. 

To evaluate the possibility that maternal depression contributed to iron deficiency, we compared iron measures in PPD (EPDS score ≥ 10) and non-PPD women (EPDS score < 10). We repeated the analysis of an EPDS total score of 12 or higher since cutoffs of 10 or 12 have been reported in previous studies [24]. General linear model (GLM) analyses were used to assess the effects controlling for covariates. To evaluate potential covariates, the relations between each background characteristic and iron status and EPDS scores were evaluated using correlations for continuous variables and Chi-square analyses for categorical variables. In the pilot sample, potentially confounding variables were considered in the initial models if they were even weakly related to either maternal iron status or EPDS score (*P* < 0.10). Due to the large sample size in the confirmatory sample, background variables were considered as potential covariates if *r* ≥ 0.10 and *P* < 0.05. Analyses within a sample were conducted in SPSS 19.0 (SPSS, Chicago, IL, USA); comparisons between samples used GraphPad QuickCalc software (GraphPad, La Jolla, CA, USA).

## 3. Results and Discussion

In the pilot sample, 137 participants had Hb from chart review and EPDS at 6 weeks postpartum. One woman who was severely anemic at both assessments (Hb < 70 g/L) was not included. In the confirmatory sample, 567 women had a complete assessment of iron status at mid pregnancy. Almost all (98%, 557/567) also had a second iron status assessment at late pregnancy. One woman with Hb concentrations >250 g/L was not included. ZPP concentrations were available for 330 women at mid pregnancy and 452 at late pregnancy. For 265 women, Hb and MCV concentrations at 3 days postpartum were available by chart review.

Background characteristics of the pilot and confirmatory samples are presented in Table 2. Infant characteristics were similar in the two samples. Women in the pilot sample were older (2.4 years), more educated (34.7% more high school graduated), and had higher annual income (40.6% more with income >5000¥) compared to the confirmatory sample. Hb concentrations in late pregnancy were similar in the pilot and confirmatory samples (*t*_(691)_ = 1.5, *P* = 0.13). As expected, a reduction in iron status was observed during pregnancy. In both samples, Hb concentrations decreased and percentage of anemia increased in late pregnancy. In the pilot sample, Hb concentrations were significantly higher (paired *t*_(134)_ = 8.6, *P* < 0.001) at early pregnancy (124.8 ± 10.9 g/L) compared to late pregnancy (113.2 ± 12.2 g/L). The percentage of anemic mothers in late pregnancy (54/135; 40%) was more than 4 times higher than in early pregnancy (12/137; 9%). We considered it highly likely that most anemia in late pregnancy in the pilot sample was due to iron deficiency, because a prior study of over 3500 pregnant women in the same rural area in China found that 87% of those with Hb < 110 g/L had ferritin < 20 ng/mL [25]. In the confirmatory sample, iron status measures at the first assessment correlated with those in late pregnancy and 3 days postpartum (*r*s ranging from 0.26 to 0.55, *P* values < 0.001). The proportion of women with anemia increased from 13% at the first assessment (74/567) to 33% in late pregnancy (186/557) and 53% after delivery (141/265). As indicated by sTfR Index >14, 30% of the anemic women were iron deficient in mid pregnancy whereas 93% were iron deficient in late pregnancy. Other iron measures in the confirmatory sample also indicated poorer iron status in late pregnancy and after delivery (Table 3). The prevalence of anemia among pregnant women in our study is similar to previous reports among pregnant women in China [2, 19].

In the pilot sample, the mean total EPDS score at 6 weeks was 7.4 ± 3.7, and the proportion of women with a total score ≥10, suggesting PPD, was 23.4% (32/137). In the confirmatory sample, EPDS data were available for 555 women at 24–28 hours after delivery; 488 had EPDS data at 6 weeks postpartum. The mean EPDS scores were similar at 24–48 hours and 6 weeks (6.7 ± 4.3 and 6.4 ± 4.1, respectively, paired *t*_(474)_ = 1.5, *P* = 0.13). The proportion of women with possible PPD was also similar (24.5% (136/555) at 24–48 hours and 20.3% (99/488) at 6 weeks (Fisher's exact test *P* = 0.06). Although the mean EPDS score in both samples was well below the cutoff for possible PPD, scores were somewhat higher in the pilot sample compared to the confirmatory sample (7.4 ± 3.7 and 6.3 ± 4.1, respectively, *t*_(623)_ = 2.9, *P* < 0.01). The proportion of women with possible PPD was similar in both samples (Fisher's exact test *P* = 0.48). The prevalence of PPD in Chinese women has ranged from 5.5% to 25.0% in previous studies, with the exception of higher prevalence among women exposed to domestic violence [23, 26–30]. The wide range appears related to background and methodological characteristics, such as socioeconomic status and education, social support, the questionnaire and timeframe used to measure postpartum depression, and severity of depression [24]. Using EPDS with a cutoff score of 10, the percentage of PPD in our study (20–23%) is within the range in other studies with Chinese women. 

EPDS scores in anemic and nonanemic women did not differ in either sample (Table 4). Furthermore, maternal iron status showed no correlation with EPDS score in either the pilot or confirmatory sample. In the pilot sample, the *r* values between maternal Hb and EPDS at 6 weeks were *r* = 0.07 and *r* = −0.01 for early and late pregnancy Hb, respectively. In the confirmatory sample, the correlations between iron measures (Hb, MCV, ZPP, ferritin, sTfR, and sTfR Index) in mid- or late pregnancy or 3 days postpartum and EPDS scores shortly after delivery or at 6 weeks were also low (*r* values < 0.10). Even in this large sample, no correlation reached statistical significance with the exception of sTfR at mid and late pregnancy and EPDS at 6 weeks (*r*s = − 0.11, *P* = 0.02). The direction of the effect, that is, higher sTfR (worse iron status) related to lower depressive symptoms, was opposite from what would be expected. 

These negative findings regarding prenatal and postpartum maternal iron status and PPD do not support a previous study linking PPD and postpartum maternal iron status as a risk factor for maternal functioning during the postpartum period [8]. The small sample size in that study might limit reproducibility of results. However, its strong treatment design provides compelling evidence of an effect of iron therapy on maternal functioning. The observational nature of our study might explain in part the differing findings. 

Women with or without possible PPD were similar in iron status in both Chinese samples. In the pilot sample, Hb concentrations in PPD (EPDS > 10) and non-PPD (EPDS ≤ 10) women averaged 124.6 ± 8.9 g/L versus 125.4 ± 11.8 g/L in early pregnancy (*F*_(1,137)_ = 0.03, *P* = 0.85; infant gender was a significant covariate) and 110.2 ± 9.5 g/L and 114.0 ± 12.8 g/L in late pregnancy (*F*_(1,132)_ = 2.02, *P* = 0.16; maternal education was a significant covariate). Iron status measures in possible PPD and non-PPD women in the confirmatory sample are compared in Table 5. The findings were the same with an EPDS cutoff of 12 (data not shown). Our findings do not support previous findings of lower ferritin concentrations in women with PPD compared to controls [15]. The higher prevalence of depression in our samples compared to the previous study (>20% versus 10%) might contribute to the different results. 

## 4. Conclusions

There were no relations between maternal iron status and maternal symptoms of postpartum depression in two independent samples from different regions of China. These negative findings are similar to results in one other large observational study [12] but differ from several studies that found an association [8, 13–15]. There are no obvious or consistent differences between our study and, previous ones in study design, timing, and measures of iron status or PPD. Moreover, the severity of anemia and the prevalence of PPD in our study are generally comparable to others. Thus, we could not identify likely reasons for the negative findings in our study, in contrast to positive findings in others. Turning to studies of iron status and depressive symptoms in women of reproductive age more broadly, methodological differences limit direct comparisons with our study. Prior studies are heterogeneous in design. About half were observational and half were interventional. Some focused on pregnant women and others on nonpregnant women. The majority of the studies were conducted in developing countries, and a variety of iron measures were used. Although several studies reported an association between iron status and depressive symptoms, some did not (see Table 1 and a review [31]). Our large study in China adds to the group of studies finding no relationship between women's iron status and depressive symptoms. Further research is needed to determine why iron status relates to depressive symptoms in some contexts but not others.

## Figures and Tables

**Table 1:** Summary of studies on maternal iron status and postpartum depression.

Country, year	Study design	Iron status	Postpartum depression	Findings
		measures and timing	measures and timing	
UK, 1994 [12]	Observational	pre- and postnatal Hb (*n* = 1010)	EPDS: 10 d, 4 wk, 6 wk	No relationship

Germany, 1995 [13]	Intervational	Postnatal: anemic treated by placebo (*n* = 36) versus anemic treated by rhEPO^a^ (*n* = 35) versus nonanemic (*n* = 274)	blues questionnaire and SCL-90-R: 5 d	Depressive symptoms: anemic > nonanemic anemic: placebo = rhEPO

US, 2003 [14]	Observational	Postnatal Hb: anemic (*n* = 8) versus nonanemic (*n* = 29)	CES-D: 28 d	depressive symptoms: anemic > nonanemic

S. Africa, 2005 [8]	Intervational	Postnatal: Hb, MCV, ferritin, sTfR IDA treated by placebo (*n* = 21) versus IDA treated by iron (*n* = 30) versus nonanemic (*n* = 30)	EPDS: 10 wk, 9 mo	depressive symptoms: IDA + iron: 10 w > 9 mo IDA + placebo: 10 wk = 9 mo

Spain, 2011 [15]	Observational	Postnatal: ferritin, transferrin, iron, sTfR	EPDS: 2 d, 8 wk, 32 wk Women with PPD at 32 wk (*n* = 65) versus non-PPD (*n* = 664)	Ferritin concentration: PPD < non-PPD

^a^rhEPO:  recombinanthumanerythropoietin.

**Table 2:** Background characteristics of the two samples in China^a^.

	Sample		
	Pilot	Confirmatory	*P* ^b^
	(*n* = 137)	(*n* = 567)	
Mother and family			
Mother age, years	27.0 ± 2.9	24.6 ± 3.7	<0.001
Mother high school graduate % (*n*)	66.2 (90/136)	31.5 (174/552)	<0.001
Annual household income % (*n*)			<0.001
<5000, *¥* ^c^	46.5 (59/127)	87.1 (481/552)	
>5000, ¥	53.5 (68/127)	12.9 (71/552)	
Infant			
Gender, %male (*n*)	53.3 (73/137)	54.5 (305/560)	0.44
Gestational age, weeks	39.6 ± 0.9	39.7 ± 1.2	0.36
Birth weight, g	3420.1 ± 452.8	3375.6 ± 426.4	0.28

^a^ *n* varies slightly due to occasional missing data for some measures. Values are expressed as means ± SD or %  (*n*) for categorical variables.

^b^ *P* values are based on *t*-tests for continuous variables and Fisher's exact analyses for categorical variables.

^c^Yuan Renminbi, sign ¥, is the official currency of China. Approximate exchange rate 1$ = 6.31¥.

**Table 3:** Maternal iron status in the confirmatory sample (mid- and late pregnancy, 3 days postpartum).

Iron measure	Mid pregnancy	Late pregnancy	3 days postpartum
	(*n* = 567)^a^	(*n* = 557)^b^	(*n* = 265)
Hemoglobin, g/L	121.5 ± 9.8	114.7 ± 9.9	104.2 ± 11.2^c^
MCV, fl	85.1 ± 4.8	84.8 ± 4.7	78.9 ± 8.3^d^
ZPP, *μ*mol/mol	54.9 ± 31.6	88.4 ± 44.2^e^	—
Ferritin, ng/mL	41.9 ± 35.3	14.1 ± 13.8^e^	—
sTfR, nmol/L	15.6 ± 5.3	30.6 ± 10.7^e^	—
sTfR Index	11.6 ± 7.4	31.9 ± 17.0^e^	

^a^ZPP values were available for 330 subjects.

^b^ZPP values were available for 452 subjects, ferritin and sTfR for 543 subjects.

^c^Significant differences were found between the 3 measures, *P* < 0.001.

^d^Significant differences were found between the 3 measures: mid pregnancy compared to late pregnancy, *P* < 0.05; mid pregnancy and late pregnancy compared to postpartum, *P* < 0.001.

^e^Significant differences were found between the 2 measures, *P* < 0.001.

**Table 4:** EPDS total scores in anemic and nonanemic mothers^a^.

	Pilot sample			Confirmatory sample		
	Anemic	Non-anemic	*P*	Anemic	Non-anemic	*P* ^b^
Early/mid pregnancy						
EPDS scale, 24–48 h				(*n* = 72)	(*n* = 483)	
				6.4 ± 4.1	6.6 ± 4.3	0.43^c^
EPDS scale, 6 wk	(*n* = 12)	(*n* = 125)		(*n* = 71)	(*n* = 417)	
	7.9 ± 6.1	7.4 ± 3.4	0.69	5.7 ± 4.4	6.4 ± 4.1	0.16
Late pregnancy						
EPDS scale, 24–48 h				(*n* = 181)	(*n* = 366)	
				7.0 ± 4.1	6.3 ± 4.3	0.10
EPDS scale, 6 wk	(*n* = 54)	(*n* = 81)		(*n* = 165)	(*n* = 315)	
	7.7 ± 4.1	7.2 ± 3.4	0.51	6.6 ± 4.3	6.1 ± 4.0	0.26
3 days postpartum						
EPDS scale, 24–48 h				(*n* = 140)	(*n* = 124)	
				7.2 ± 3.9	7.2 ± 4.1	0.48
EPDS scale, 6 wk				(*n* = 130)	(*n* = 118)	
				5.9 ± 4.0	6.4 ± 4.0	0.40

^a^Values are unadjusted means ± SD.

^b^ *P* values are based on GLM analyses with covariate control as indicated.

^c^Maternal education was a significant covariate.

**Table 5:** Iron status in mothers with possible PPD and non-PPD in the confirmatory sample^a^.

EPDS	24–48 hours			6 weeks		
	Possible PPD	Non-PPD	*P* ^b^	Possible PPD	Non-PPD	*P* ^b^
Mid pregnancy	(*n* = 136)	(*n* = 418)		(*n* = 99)	(*n* = 389)	

Hemoglobin g/L	121.2 ± 9.5	121.5 ± 9.9	0.76	120.5 ± 9.6	121.3 ± 10.3	0.49
MCV, fl	85.2 ± 3.6	85.1 ± 5.1	0.77	85.6 ± 3.5	85.1 ± 5.2	0.41
ZPP, *μ*mol mol^c^	58.5 ± 39.9	54.2 ± 29.2	0.41^d^	61.0 ± 42.7	56.5 ± 29.8	0.32
Ferritin, ng/mL	41.4 ± 32.5	41.5 ± 35.8	0.84^e^	43.7 ± 30.9	42.9 ± 36.9	0.83^e^
sTfR, nmol/L	15.4 ± 4.6	15.7 ± 5.5	0.52^d–g^	14.3 ± 3.8	15.9 ± 5.7	0.02^e–g,i^
sTfR Index	11.8 ± 8.2	11.8 ± 7.7	0.82^f,g^	10.5 ± 6.5	11.9 ± 7.8	0.12^g^

Late pregnancy	(*n* = 134)	(*n* = 413)		(*n* = 96)	(*n* = 384)	

Hemoglobin g/L	113.9 ± 9.7	115.0 ± 10.0	0.27^e^	114.0 ± 9.2	114.4 ± 10.0	0.73
MCV, fl	84.6 ± 3.9	84.9 ± 4.9	0.56	85.4 ± 3.8	84.7 ± 4.8	0.15
ZPP, *μ*mol mol^c^	88.4 ± 41.1	88.5 ± 45.4	0.98	91.1 ± 47.8	91.9 ± 44.9	0.89
Ferritin, ng/mL	14.8 ± 16.5	13.7 ± 12.8	0.61	14.3 ± 11.3	14.0 ± 14.6	0.87
sTfR, nmol/L	29.4 ± 9.7	31.0 ± 10.9	0.11	30.0 ± 10.2	30.9 ± 10.8	0.50
sTfR Index	30.2 ± 14.4	32.5 ± 17.9	0.20	31.6 ± 16.9	32.3 ± 17.5	0.72

3-days postpartum	(*n* = 69)	(*n* = 195)		(*n* = 44)	(*n* = 204)	

Hemoglobin g/L	103.3 ± 12.2	104.6 ± 10.8	0.62^e^	103.5 ± 11.2	104.5 ± 11.4	0.51^e^
MCV, fl	77.6 ± 11.7	79.3 ± 6.7	0.07^h^	77.2 ± 12.0	79.4 ± 6.6	0.09^h^

^a^ *n* varies slightly due to occasional missing data for some measures. Values are unadjusted means ± SD.

^b^ *P* values are based on GLM analyses controlling for significant covariates as indicated by d–h.

^c^At 24–48 hours, ZPP values in mid pregnancy are available for 330 subjects, 70 possible PPD and 250 non-PPD, and for late pregnancy are available for 442 subjects, 101 possible PPD and 341 non-PPD. At 6 weeks, ZPP values in mid pregnancy are available for 268 subjects, 60 possible PPD and 208 non-PPD, and for late pregnancy are available for 378 subjects, 78 possible PPD, and 300 non-PPD.

^d^Maternal education, ^e^birth weight, ^f^maternal age, ^g^gestational age, and ^h^annual income were significant covariates.

^i^Note that the direction of effect is higher sTfR (worse iron status) in the non-PPD group.

## Abbreviations

Hb: Hemoglobin
MCV: Mean corpuscular volume
ZPP: Zinc protoporphyrin
PPD: Postpartum depression
EPDS: Edinburgh Postnatal Depression Scale
sTfR: Soluble transferrin receptor
sTfR Index: soluble transferrin receptor/log ferritin index.
